# Supplementary material for: Hemagglutinin Gene Variation Rate of H9N2 Avian Influenza Virus by Vaccine Intervention in China
Source: Viruses. 2022 May 13;14(5):1043. doi: 10.3390/v14051043 (PMC9146883; doi:10.3390/v14051043)
Supplement: Supplementary file 1 [file viruses-14-01043-s001.zip › viruses-1671820-suppl.pdf]

viruses

MDPI

Table S1. Basic information of H9N2 avian influenza vaccine strains in China.

No	Abbreviation	Strains	Analyzed	Approval date
1	F	A/Chicken/Shanghai/F/98	Yes	2002.4
2	SS	A/Chicken/Guangdong/SS/94	Yes	2003.9.2
3	SD696	A/Chicken/Shandong/6/96	Yes	2004.1.19
4	HL	A/Chicken/Henan Luo-yang/HL/2001	Yes	2006.9.30
5	SY	A/Chicken/Shanxi/SY/97	Yes	2006.4.25
6	SS/94	A/Chicken/Guangdong/ss/94	Yes	2007.6.1
7	Re-2	A/chicken/Guangxi/10/1999	Yes	2007.2.9
8	YBF003	A/Chicken/Shandong/11/05	Yes	2009.7.21
9	WD	A/Chicken/Hebei/WD/98	Yes	2009.3.6
10	HP	A/Chicken/Henan Puyang/2/98	Yes	2009.3.6
11	L	A/Chicken/Shandong/1/2002	Yes	2009.3.6
12	HN106	A/Chicken/Henan/01/2006	Yes	2010.12.7
13	JY	A/Chicken/Jiangsu/JY/99	Yes	2010.11.30
14	NJ02	A/Chicken/NanJing/02/2001	Yes	2010.8.27
15	H2	/	No	/
16	LG1	A/Chicken/Shandong/LG1/2000	No	2010.1.11
17	S2	A/Chicken/Shandong/S2/2005	Yes	2011.1.21
18	HZ	A/Chicken/Henan Zheng-zhou/HZ/2001	Yes	2011.3.18
19	NJ01	A/Duck/Nanjing/01/1999	Yes	2013.5.6
20	SZ	A/Chinken/shanghai/10101	Yes	2015.6.25
21	Re-9	/	No	2015.11.18
22	JD	A/Chicken/Shandong/JD/2010	Yes	2017.8.31
23	HN03	A/Chicken/Henan/03/2009	No	2017.5.23
24	WJ57	A/Chicken/Hebei/WJ57/2012	Yes	2018.10.15
25	G	A/Chicken/Hebei/G/2012	Yes	2019.4.16
26	YBF13	/	Yes	2021.6.29
27	YT	A/Chicken/Hebei/YT/2010	No	2021.8.29
28	SH	/	No	/

*"/" indicates that information is not available.

Table S2. Detailed distribution of H9N2 subtype AIVs in different countries or cities.

Global Countries	Counts	Provinces in mainland China	Counts
China	1461	Guangdong	195
Vietnam	282	Jiangxi	175
Bangladesh	134	Shandong	162
United States	82	Jiangsu	161
Cambodia	64	Zhejiang	150
Pakistan	55	Hunan	121
Egypt	53	Hong Kong	59
Israel	42	Shanghai	54
Japan	38	Guangxi	51
Laos	25	Anhui	42
Jordan	18	Fujian	41
South Korea	17	Hubei	41
Netherlands	12	NA	41
Saudi Arabia	10	Jilin	22
Iran	8	Hebei	21
Afghanistan	7	Beijing	20
Chile	6	Yunnan	20
India	5	Henan	12
Russia	5	Sichuan	8
Sweden	5	Chongqing	6
Argentina	3	Shanxi	4
Belgium	3	Gansu	3
Canada	3	Guizhou	3
Ghana	3	Liaoning	3
Iraq	3	Shaanxi	3
Morocco	3	Tianjin	3
Poland	3	Tibet	3
Portugal	3	Heilongjiang	2
Lebanon	2	NeiMenggu	2
United Arab Emirates	2	Ningxia	2
Burkina Faso	1	Hainan	1
Germany	1	Oman	1
Indonesia	1	Xinjiang	1
Malaysia	1	Qinghai	0
Mexico	1	/	/
NA	1	/	/

*"/" indicates that information is not available.
